# Supplementary material for: A multidimensional analysis reveals distinct immune phenotypes and the composition of immune aggregates in pediatric acute myeloid leukemia
Source: Leukemia. 2024 Aug 26;38(11):2332–43. doi: 10.1038/s41375-024-02381-w (PMC11518988; doi:10.1038/s41375-024-02381-w)
Supplement: Supplementary file 1 — supplementary information including methods, figures and legends [file 41375_2024_2381_MOESM1_ESM.pdf]

Supplementary Information

to

A multidimensional analysis reveals distinct immune phenotypes and the composition of immune aggregates in pediatric acute myeloid leukemia

Koedijk et al.

Supplementary Methods

Ethical regulation

Pediatric AML bone marrow (BM) biopsy tissues acquired from external biobanks (n=28) were leftover material from standard care procedures and therefore, no informed consent was acquired, according to Dutch legislation and the code of conduct of the Committee on Regulation of Health Research (COREON).

Human patient samples

As non-leukemic controls, FFPE BM biopsies from age- and sex-matched children with treatment-naïve early-stage rhabdomyosarcoma were obtained from the Princess Máxima Center Biobank (n=10). An experienced hemato-onco pathologist confirmed that these control biopsies resembled normal hematopoiesis and that there was no malignancy infiltrating the BM. Diagnostic flow cytometry reports from an independent cohort of 20 pediatric cases with normal karyotype AML, all treated at the Princess Máxima Center, were utilized to investigate the BM T cell abundance (% of live cells) in *FLT3*-ITD and/or *NPM1*-mutated cases compared to wildtype cases (no molecular aberrations identified). AML patients were all treated using intensive anthracycline- and cytarabine-based chemotherapy and allogeneic stem cell transplantation in selected high-risk cases[1].

Response definitions of ETCTN 10026 clinical trial in adult AML patients

We used the definitions of response according to the 2003 International Working Group criteria for acute myeloid leukemia[2]. All responders achieved a morphologic CR. Among the non-responders (AML1010, AML1011, AML1003), AML1010 had >5% blasts. Although AML1011 had <5% blasts in the aspirate, histology indicated 5% blasts. Furthermore, AML1003 had <5% blasts but also an aplastic marrow without hematologic recovery, which was classified as morphologic leukemia-free state (MLFS; part of the non-response categories).

Immunohistochemistry/immunofluorescence and digital image analysis

BM pediatric and adult AML cases were cut into consecutive sections of 4 and 5 μm, respectively. Conventional IHC was performed for CD3 (T cells), CD3-CD4 (duplex; for $CD4^+$ T cells and $CD4^+$ AML cells), CD8 (mainly $CD8^+$ T cells but may also stain rare $CD8^+$ NK- and dendritic cells), CD20 (B cells), CD34, CD117, and CD15 (immunophenotype-based AML markers) on pediatric AML BM biopsies using a Ventana Benchmark Ultra (Roche, Basel, Switzerland) automated staining instrument according to manufacturers' recommendations. A list of antibodies and suppliers is available in **Table S5**. Digital scans of stained slides were obtained using a NanoZoomer scanner (Hamamatsu, Shizuoka, Japan).

Multiplex immunofluorescence using DAPI and antibodies against CD3 (T cells), CD8 ($CD8^+$ T cells), granzyme B (GZMB; cytotoxicity marker), PD-1, TIM-3, and LAG3 (all immune checkpoint receptors) was performed on two treatment-naïve pediatric AML biopsies (AML2 + AML6) using SignalStar™ from Cell Signaling Technology as previously described[3]. Briefly, CD3, CD8, granzyme B, PD-1, TIM-3, and LAG3 were conjugated to oligonucleotides (oligos) and then validated in the SignalStar multiplex assay to assess the ($CD8^+$) T cell compartment of the tumor microenvironment (a list of antibodies and suppliers is listed in **Table S5**). All primary antibodies were applied at once in one primary incubation step. Complimentary oligos with fluorescent dyes (channels: 488, 594, 647, and 750 nm) subsequently amplified the signal of up to four oligo-conjugated antibodies in the first round of imaging. Then, the first round of oligos and fluorophores were gently removed and a second batch of complementary oligos with fluorescent dyes was added to again amplify the signal of up to four additional oligo-conjugated antibodies. Staining of the biopsies was done on a BOND RX fully automated stainer (Leica Biosystems, Nußloch, Germany) after slide baking for 30 minutes at 60°C. Imaging was performed using a Leica DMi8 inverted microscope (Leica Camera AG, Wetzlar, Germany), including automated stage and 3D Thunder deconvolution, using a 20x dry objective equipped with a Leica DFC700GT camera. Slides were scanned and images were deconvoluted and stitched using LASX (Leica). Subsequently, images were analyzed in QuPath[4] as described below. Multiplex immunofluorescence using DAPI and antibodies against CD3 (T cells) and FOXP3 (putative regulatory T cell marker) was performed on the same treatment-naïve pediatric AML biopsies (AML2 + AML6; consecutive slides) using the Ventana Benchmark Discovery (Ventana Medical Systems Inc; a list of antibodies and suppliers is provided in **Table S5**) and scanned (ZEISS Axioscan 7; Zeiss, Oberkochen, Germany) as previously described[5].

Multiplex immunofluorescence using DAPI and antibodies against CD3 (T cells), CD20 (B cells), and CD34 (AML blasts and endothelial cells) was performed on baseline, time of best response, and end of treatment (EOT) BM biopsies of adult AML cases treated with ipilimumab-based therapy on the 10026 study and imaged as previously described[2]. The time points analyzed for each patient were based on the availability of BM biopsies**.** One biopsy was excluded because of poor quality [EOT case AML1003]. Staining of the biopsies was completed on a BOND RX fully automated stainer (Leica Biosystems, Nußloch, Germany) after baking tissue sections for 3 hours at 60°C. The BOND RX then performed deparaffinization and rehydration with series of graded ethanol to deionized water. Subsequently, antigen retrieval was performed utilizing Epitope Retrieval Solution 1 (pH 6) or 2 (pH 9), as shown in **Table S6** (ER1, ER2, Leica Biosystems, Cat. AR9961, AR9640). The secondary antibodies that were used are described in **Table S6** as well. The antibody complex

was incubated for 10 minutes with its corresponding Opal Fluorophore Reagent (Akoya Biosciences, Marlborough, MA, USA) for signal visualization. After the final fluorophore application, the samples were incubated in Spectral DAPI solution (Akoya) for 10 minutes. Lastly, samples were removed from the BOND, air-dried, and mounted using Prolong Diamond Anti-fade mounting medium (Life Technologies, Cat. P3695). Whole-slide images have been obtained using the PhenoImager HT multispectral imaging platform (Akoya), were spectrally unmixed (Inform 2.6, Akoya), and 20x regions were stitched together (QuPath, v0.3.2.)[3]. For all imaged slides, whole-slide digital image analysis was performed in QuPath[3]. Inside QuPath, the deep learning-based cell segmentation tool StarDist and the machine learning-based Random Trees classifier were used to quantify the number of positive cells per mm^2 [4, 6]. T cell proximity was established using Delaunay Triangulation[7].

Immune-related gene expression profiling

Four consecutive sections of 10 µm from six immune-infiltrated and seventeen immune-depleted FFPE BM biopsies from a cytogenetically representative cohort of pediatric AML cases (patient characteristics in **Table S1**) were used to isolate RNA and to perform immune-related gene expression profiling with the 770-gene PanCancer IO 360 panel, as previously described (NanoString, Seattle, WA, USA)[8, 9]. After passing quality control, raw data were normalized using ROSALIND® according to NanoString's recommendations (https://rosalind.bio/; San Diego, CA, USA). Normalized data were uploaded to the online iDEP platform and differentially expressed genes between immune-infiltrated and immune-depleted biopsies were identified using *DEseq2* with a false-discovery rate (FDR) cut-off of 0.05 and a minimum fold change of 2 (integrative Differential Expression and Pathway analysis; http://bioinformatics.sdstate.edu/idep96/; V.0.96)[10]. Pathway analysis was performed using single-sample gene set enrichment analysis with the GO Biological Processes and WikiPathways gene sets with an FDR cut-off of 0.05[11-13]. The M2-predominance score was estimated using a published gene signature of M2-like macrophages compared to M1-like macrophages, inside the TIDE environment (tide.dfci.harvard.edu/)[14, 15].

GeoMx Digital Spatial Profiling

5 µm thick FFPE BM biopsy sections from six pediatric AML cases with an immune-infiltrated BM and two non-leukemic controls were put on three different slides and prepared for GeoMx Digital Spatial Profiling (DSP; NanoString), as previously described[16]. Slides were simultaneously incubated with immunofluorescent antibodies and GeoMx Whole Transcriptome Atlas profiling reagents. SYTO13 (S7575, Thermo Fisher,) was used for identification of nuclei, CD45 (NBP2-34528, Novus) for leukocytes, and CD3 (NBP2-54392AF647, Novus) for T cells. Stained slides were loaded onto the GeoMx instrument and

scanned. ROIs were selected using the above-mentioned antibodies in combination with overlayed images of CD20, CD34, CD3-CD4 (duplex), and CD117 (IHC). Then, UV-photocleaved oligonucleotides were collected in separate wells and sequenced on the Nextseq2000 (Illumina, San Diego, CA, USA).

Raw data were normalized using Quartile 3 count (Q3) normalization in R (V.4.2.1) as per NanoString's recommendations (code is available in the vignette of the *Geomxtools* package: https://bioconductor.org/packages/release/workflows/vignettes/GeoMxWorkflows/inst/doc/GeomxTools_RNA-NGS_Analysis.html). Batch correction was performed using Combat-seq[17]. Spatial Deconvolution was performed using the safeTME reference (*SpatialDecon* package; cell reference profiles are available via https://github.com/Nanostring-Biostats/CellProfileLibrary/blob/archive/safeTME-for-tumor-immune.csv). Furthermore, we retrieved single-cell RNA-sequencing data from pediatric tonsillar B cells[18] (sample BC005 was chosen since it had the highest number of cells, as done previously[19]) and adult AML bone marrow $CD8^+$ T cells[20] (all patients), and converted these two additional reference profiles using R (V4.2.1). Deconvoluted abundance scores were normalized for ROI-size and, in case of immune aggregates, for the ROI-area covered by these aggregates. The '12chem', 'Tfh', and 'TLS imprint', and M2-predominance signatures were applied to Q3-normalized data and further normalized as mentioned above[19, 21-23].

External pediatric AML data

Bulk RNA-seq data (counts and transcripts-per-million; TPM) and matching clinical data of 159 treatment-naïve *de novo* pediatric AML cases generated as part of the TARGET-AML project was acquired via https://www.cBioportal.org[24, 25]. TPM values were used to estimate the abundance of total T- and $CD8^+$ T cells using CIBERSORTx, as described previously (LM22 reference profile; https://cibersortx.stanford.edu/)[26-28].

Statistical analysis

In case of no Gaussian distribution, differences between two independent groups and two paired groups were compared using the Mann-Whitney test and the Wilcoxon paired signed-rank test, respectively. For Gaussian-distributed data, unpaired and paired t-tests, respectively, were used. The correlation between two variables was evaluated using Spearman's *r*. To measure the area under the receiver operating characteristic curve, we employed the Wilson-Brown method. To estimate the survival from diagnosis, the Kaplan-Meier's methodology was employed. To assess the difference between survival estimates in different groups, the log rank test was used. In case of multiple comparisons and no Gaussian distribution of residuals, we employed the Kruskal-Wallis test followed by Dunn's test for

multiple comparisons including a Bonferroni correction. In case multiple p-values are shown, the upper one is associated with the Kruskal-Wallis test, while the lower one(s) reflect(s) the result of Dunn's multiple comparison test.

Supplementary references

1. de Rooij JD, Zwaan CM, van den Heuvel-Eibrink M. Pediatric AML: From Biology to Clinical Management. J Clin Med. 2015;4(1):127-49.
2. Cheson BD, Bennett JM, Kopecky KJ, Büchner T, Willman CL, Estey EH, et al. Revised recommendations of the International Working Group for Diagnosis, Standardization of Response Criteria, Treatment Outcomes, and Reporting Standards for Therapeutic Trials in Acute Myeloid Leukemia. J Clin Oncol. 2003;21(24):4642-9.
3. Papalegis D, Tkachev S, Vu L, Klein S. 114 SignalStar™ is a novel multiplex IHC technology that demonstrates flexibility and reproducibility. Journal for ImmunoTherapy of Cancer. 2023;11(Suppl 1):A129-A.
4. Bankhead P, Loughrey MB, Fernández JA, Dombrowski Y, McArt DG, Dunne PD, et al. QuPath: Open source software for digital pathology image analysis. Scientific Reports. 2017;7(1):16878.
5. Zhang W, Hubbard A, Jones T, Racolta A, Bhaumik S, Cummins N, et al. Fully automated 5-plex fluorescent immunohistochemistry with tyramide signal amplification and same species antibodies. Laboratory Investigation. 2017;97(7):873-85.
6. Schmidt U, Weigert M, Broaddus C, Myers G, editors. Cell detection with star-convex polygons. Medical Image Computing and Computer Assisted Intervention–MICCAI 2018: 21st International Conference, Granada, Spain, September 16-20, 2018, Proceedings, Part II 11; 2018: Springer.
7. Delaunay B. Sur la sphère vide. A la mémoire de Georges Voronoï. Известия Российской академии наук Серия математическая. 1934(6):793-800.
8. Danaher P, Warren S, Dennis L, D'Amico L, White A, Disis ML, et al. Gene expression markers of tumor infiltrating leukocytes. Journal for immunotherapy of cancer. 2017;5:1-15.
9. Vadakekolathu J, Minden MD, Hood T, Church SE, Reeder S, Altmann H, et al. Immune landscapes predict chemotherapy resistance and immunotherapy response in acute myeloid leukemia. Sci Transl Med. 2020;12(546).
10. Ge SX, Son EW, Yao R. iDEP: an integrated web application for differential expression and pathway analysis of RNA-Seq data. BMC bioinformatics. 2018;19:1-24.
11. Ashburner M, Ball CA, Blake JA, Botstein D, Butler H, Cherry JM, et al. Gene ontology: tool for the unification of biology. Nature genetics. 2000;25(1):25-9.
12. The Gene Ontology resource: enriching a GOld mine. Nucleic acids research. 2021;49(D1):D325-D34.
13. Martens M, Ammar A, Riutta A, Waagmeester A, Slenter DN, Hanspers K, et al. WikiPathways: connecting communities. Nucleic acids research. 2021;49(D1):D613-D21.
14. Jiang P, Gu S, Pan D, Fu J, Sahu A, Hu X, et al. Signatures of T cell dysfunction and exclusion predict cancer immunotherapy response. Nat Med. 2018;24(10):1550-8.
15. Beyer M, Mallmann MR, Xue J, Staratschek-Jox A, Vorholt D, Krebs W, et al. High-resolution transcriptome of human macrophages. 2012.
16. Merritt CR, Ong GT, Church SE, Barker K, Danaher P, Geiss G, et al. Multiplex digital spatial profiling of proteins and RNA in fixed tissue. Nat Biotechnol. 2020;38(5):586-99.
17. Zhang Y, Parmigiani G, Johnson WE. ComBat-seq: batch effect adjustment for RNA-seq count data. NAR genomics and bioinformatics. 2020;2(3):lqaa078.

18. King HW, Orban N, Riches JC, Clear AJ, Warnes G, Teichmann SA, et al. Single-cell analysis of human B cell maturation predicts how antibody class switching shapes selection dynamics. Sci Immunol. 2021;6(56).
19. Meylan M, Petitprez F, Becht E, Bougoüin A, Pupier G, Calvez A, et al. Tertiary lymphoid structures generate and propagate anti-tumor antibody-producing plasma cells in renal cell cancer. Immunity. 2022;55(3):527-41.e5.
20. Desai PN, Wang B, Fonseca A, Borges P, Jelloul FZ, Reville PK, et al. Single-Cell Profiling of CD8+ T Cells in Acute Myeloid Leukemia Reveals a Continuous Spectrum of Differentiation and Clonal Hyperexpansion. Cancer Immunol Res. 2023:Of1-of18.
21. Fridman WH, Meylan M, Pupier G, Calvez A, Hernandez I, Sautès-Fridman C. Tertiary lymphoid structures and B cells: An intratumoral immunity cycle. Immunity. 2023;56(10):2254-69.
22. Coppola D, Nebozhyn M, Khalil F, Dai H, Yeatman T, Loboda A, et al. Unique ectopic lymph node-like structures present in human primary colorectal carcinoma are identified by immune gene array profiling. Am J Pathol. 2011;179(1):37-45.
23. Gu-Trantien C, Loi S, Garaud S, Equeter C, Libin M, de Wind A, et al. $CD4^{+}$ follicular helper T cell infiltration predicts breast cancer survival. J Clin Invest. 2013;123(7):2873-92.
24. Bolouri H, Farrar JE, Triche T, Jr., Ries RE, Lim EL, Alonzo TA, et al. The molecular landscape of pediatric acute myeloid leukemia reveals recurrent structural alterations and age-specific mutational interactions. Nat Med. 2018;24(1):103-12.
25. Cerami E, Gao J, Dogrusoz U, Gross BE, Sumer SO, Aksoy BA, et al. The cBio cancer genomics portal: an open platform for exploring multidimensional cancer genomics data. Cancer discovery. 2012;2(5):401-4.
26. Newman AM, Steen CB, Liu CL, Gentles AJ, Chaudhuri AA, Scherer F, et al. Determining cell type abundance and expression from bulk tissues with digital cytometry. Nat Biotechnol. 2019;37(7):773-82.
27. Wang Y, Cai YY, Herold T, Nie RC, Zhang Y, Gale RP, et al. An Immune Risk Score Predicts Survival of Patients with Acute Myeloid Leukemia Receiving Chemotherapy. Clin Cancer Res. 2021;27(1):255-66.
28. Penter L, Liu Y, Wolff JO, Yang L, Taing L, Jhaveri A, et al. Mechanisms of response and resistance to combined decitabine and ipilimumab for advanced myeloid disease. Blood. 2023;141(15):1817-30.

Supplemental information figures, titles, and legends

Table Legends (tables are provided in a separate excel file)

Supplementary Table 1. Clinical characteristics of the primary study cohort, supplemented with information on immune-infiltration, performed assays, and T cell networks.

Supplementary Table 2. List of significantly up- or downregulated pathways comparing immune-infiltrated versus immune-depleted cases using ssGSEA.

Supplementary Table 3. Clinical characteristics of the adult AML cohort treated with ipilimumab-based therapy.

Supplementary Table 4. Differentially expressed genes (DEGs) between regions in the spatial transcriptomics (tx) dataset identified by linear mixed-effect modelling (LMM).

Supplementary Table 5. Target antigens, antibody clones and suppliers of antibodies used for pediatric AML bone marrow IHC/multiplex immunofluorescence.

Supplementary Table 6. Target antigens, antibody clones, suppliers, dilution of markers, diluents, and antigen retrieval conditions used for adult AML bone marrow multiplex immunofluorescence.

Supplementary Figure 1

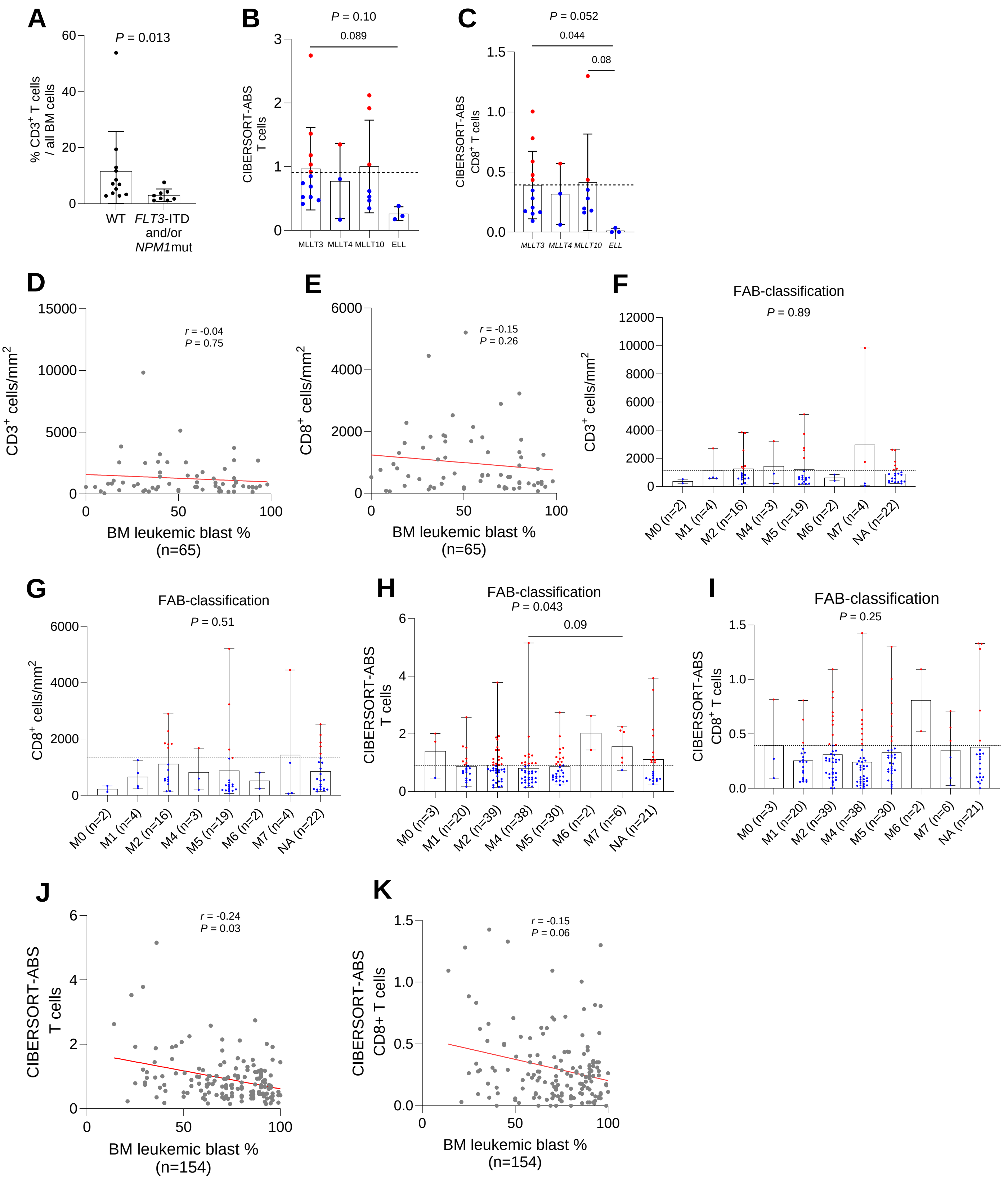

Supplementary Figure 1. AML blasts and differentiation stage in relation to T- and CD8$^+$ T cell infiltration in the bone marrow. (A) Comparison of the percentage of CD3$^+$ T cells out of all bone marrow (BM) cells between diagnostic pediatric AML cases with normal karyotype and no identified molecular aberrations (wildtype) and those with a *FTL3*-ITD and/or *NPM1* mutation (*NPM1*mut; Mann-Whitney test). The T cell proportions were retrieved from diagnostic flow cytometry reports. These patients are part of an independent cohort, not the primary study cohort. (B-C) Comparison of the CIBERSORTx-based estimated absolute (ABS) abundance of T- (B) and CD8$^+$ T cells (C) between pediatric AML patients (TARGET-AML cohort) with different *KMT2A*-rearrangements. The names on the x-axis indicate the *KMT2A*-fusion partners. The dashed lines indicate the median estimated abundance of T- and CD8$^+$ T cells in four non-leukemic controls. Examined using the Kruskal-Wallis test followed by Dunn's multiple comparisons test. In case of multiple p-values, the upper one is associated with the Kruskal-Wallis test, while the lower one(s) reflect(s) the result of Dunn's multiple comparison test. (D-E) Correlation plot between the normalized number of CD3$^+$ (D) and CD8$^+$ (E) T cells and leukemic blasts in the BM (blast % available for 65 cases), calculated using Spearman correlation. (F-G) Comparison of the normalized abundance of CD3$^+$ (F) and CD8$^+$ (G) T cells in the BM across AML differentiation stages (FAB-classifications; Kruskal-Wallis followed by Dunn's multiple comparisons test). Data are presented as mean plus range. The dashed lines indicate the median T- and CD8$^+$ T cell abundance in non-leukemic controls. (H-I) Comparison of the estimated abundance of T- (H) and CD8$^+$ (I) T cells in the bone marrow of TARGET-AML cases across AML differentiation stages (FAB-classifications; Kruskal-Wallis followed by Dunn's multiple comparisons test). Data are presented as mean plus range. The dashed lines indicate the estimated median T- and CD8$^+$ T cell abundance in non-leukemic controls. (J-K) Correlation plot between the estimated number of T- (J) and CD8$^+$ (K) T cells and leukemic blasts in the bone marrow (blast % available for 154 cases) of TARGET-AML cases, calculated using Spearman correlation.

Supplementary Figure 2

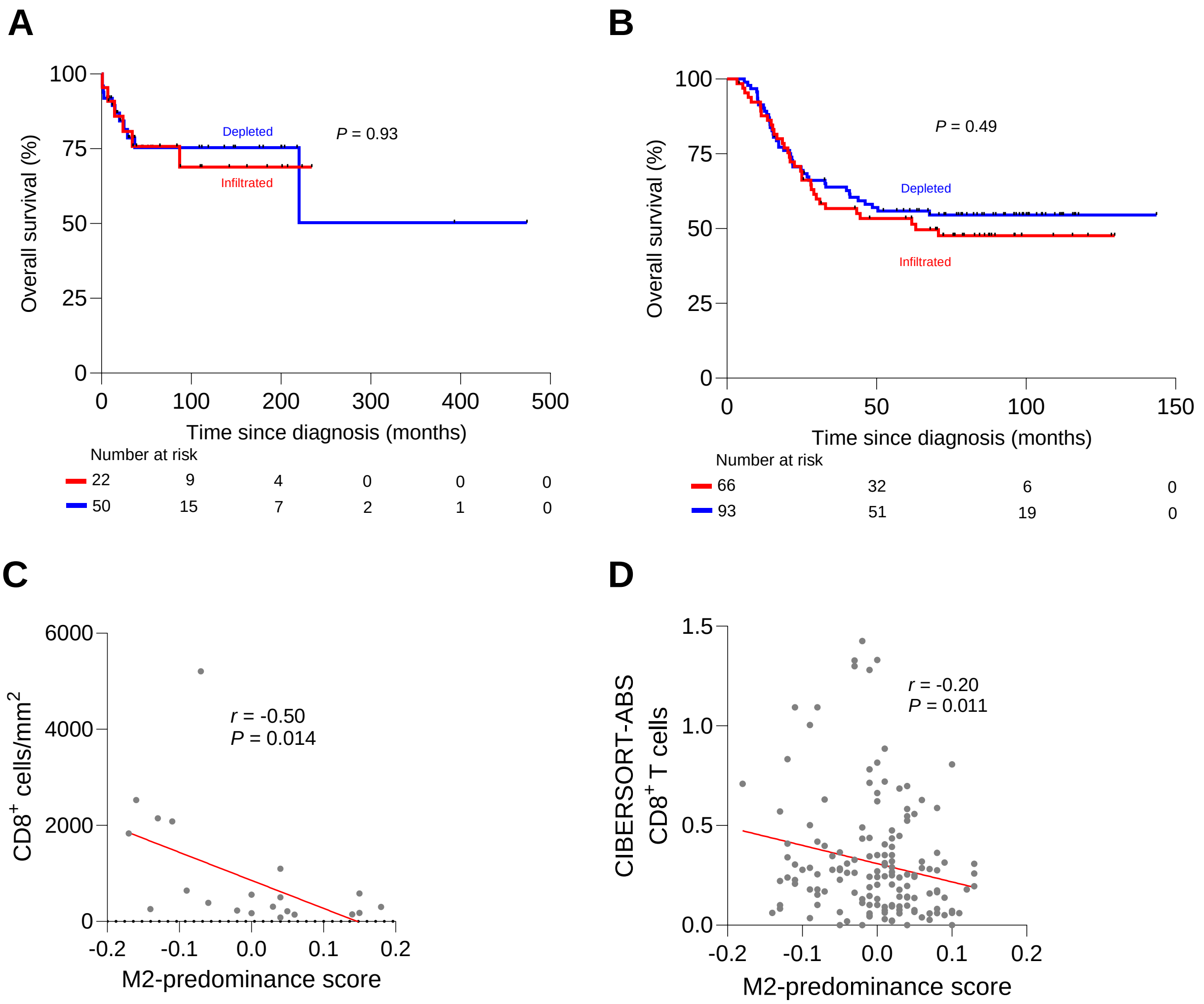

Supplementary Figure 2. Overall survival and M2-like macrophage abundance in immune-infiltrated and immune-depleted cases across two cohorts.

(A-B) Overall survival between immune-infiltrated and immune-depleted pediatric AML cases in the primary study cohort (A) and in the TARGET-AML cohort (B). (C-D) Correlation plots of the correlation between the M2-predominance score and the normalized (C; our cohort) or estimated (D; TARGET-AML cohort) abundance of $CD8^+$ T cells, calculated using Spearman correlation.

Supplementary Figure 3

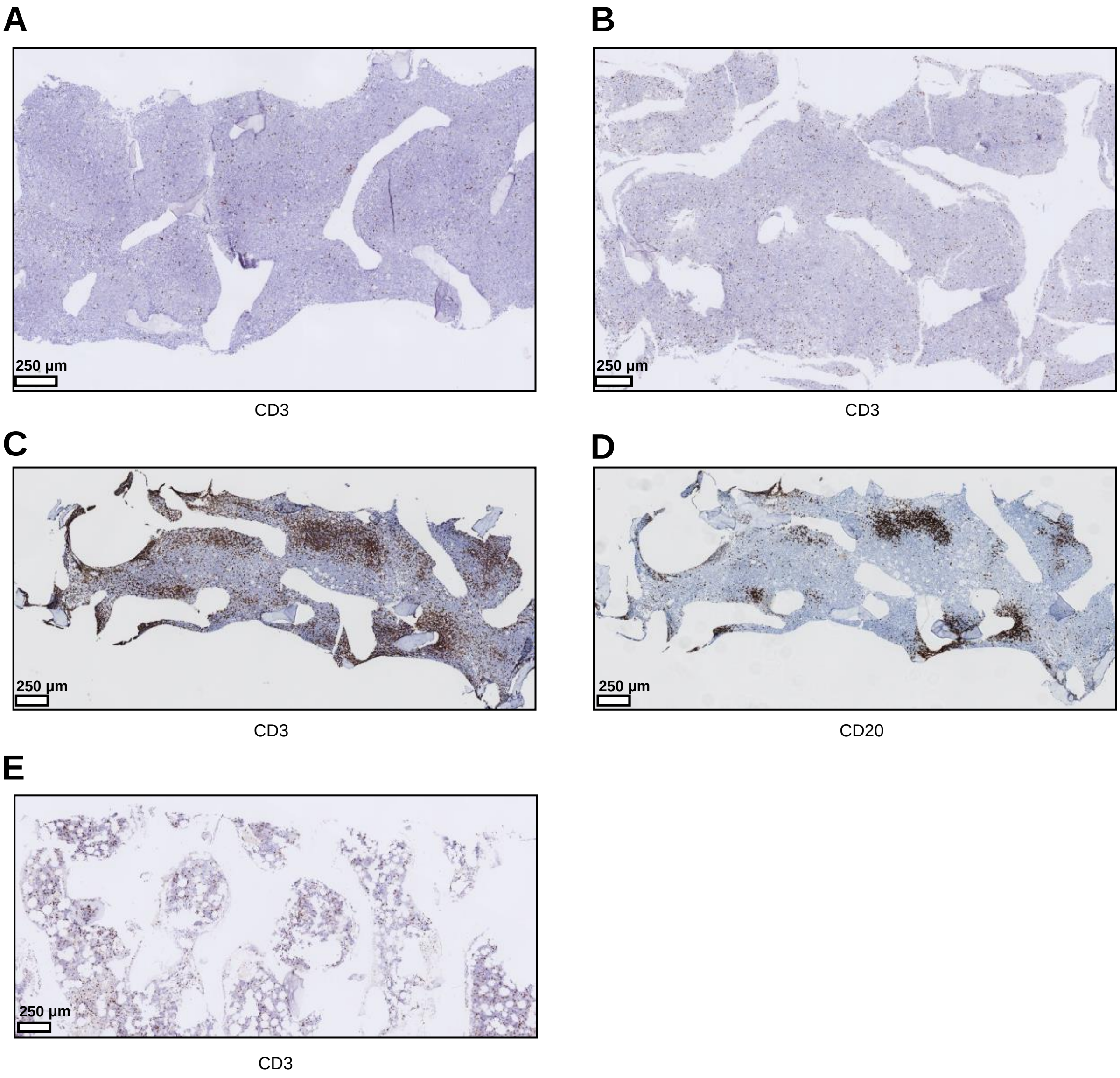

Supplementary Figure 3. Representative images of bone marrow biopsies of immune-infiltrated and immune-depleted pediatric AML, and a non-leukemic control.

(A-B) Representative images of $CD3^+$ T cells in the bone marrow (BM) of immune-depleted cases. (C-D) Representative images of large T cell networks (C) that colocalized with a dense network of B cells (lymphoid aggregates) (D). (E) Representative image of $CD3^+$ T cell infiltration in the BM of a non-leukemic control.

Supplementary Figure 4

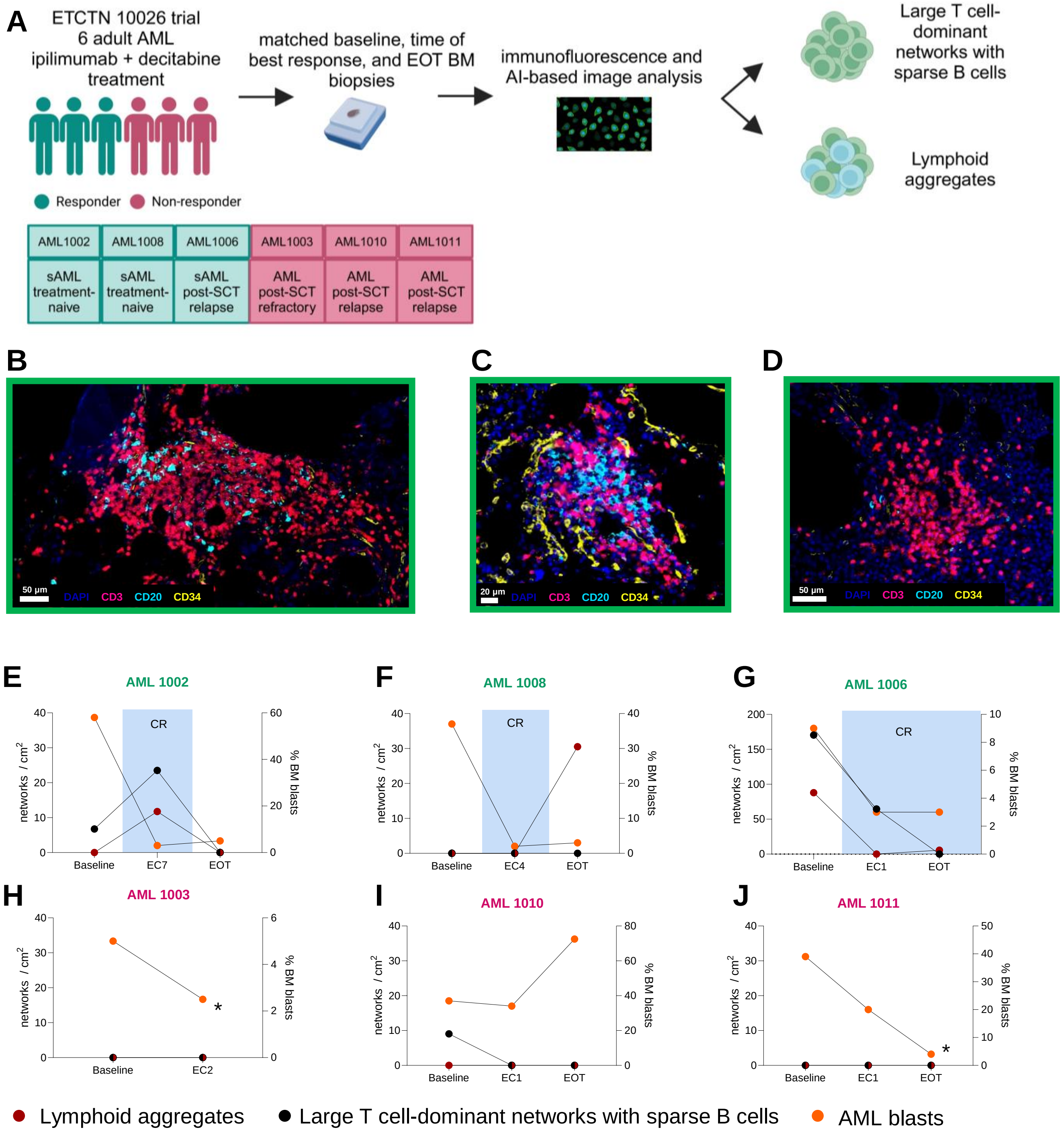

Supplementary Figure 4. Immune aggregates in the bone marrow of adult AML cases treated with ipilimumab-based treatment. (A) Schematic overview of adult AML cases that were treated with ipilimumab and decitabine, the used techniques, and the examined variables. The table indicates the patient IDs, whether patients responded (yes=green, purple=no) to these therapies, whether the patients had primary AML (AML) or secondary AML (sAML), and whether they were treated in the treatment-naïve or post-stem cell transplantation (SCT) setting. AI: artificial intelligence. BM: bone marrow; EOT: end of treatment. (B-D) Representative multiplex immunofluorescence images of lymphoid aggregates (B-C) and large T cell-dominant networks with sparse B cells (D). (E-J) Longitudinal overview of lymphoid aggregates, T cell-dominant networks, and AML blasts in BM biopsies before treatment according to the clinical trial (baseline), at time of best response, and/or at EOT. Green IDs indicate responders, purple IDs indicate non-responders. The AML blast percentage was the average of the percentages in the aspirate and the core biopsy. CR: complete morphologic remission (see supplementary methods for response criteria). EC: end of course; the number (e.g., EC1) indicates which course was given (e.g., EC1 indicates that the biopsy was taken after course 1). *AML1003 had an aplastic bone marrow without hematologic recovery, and AML1011 had 5% blasts by histology.

Supplementary Figure 5

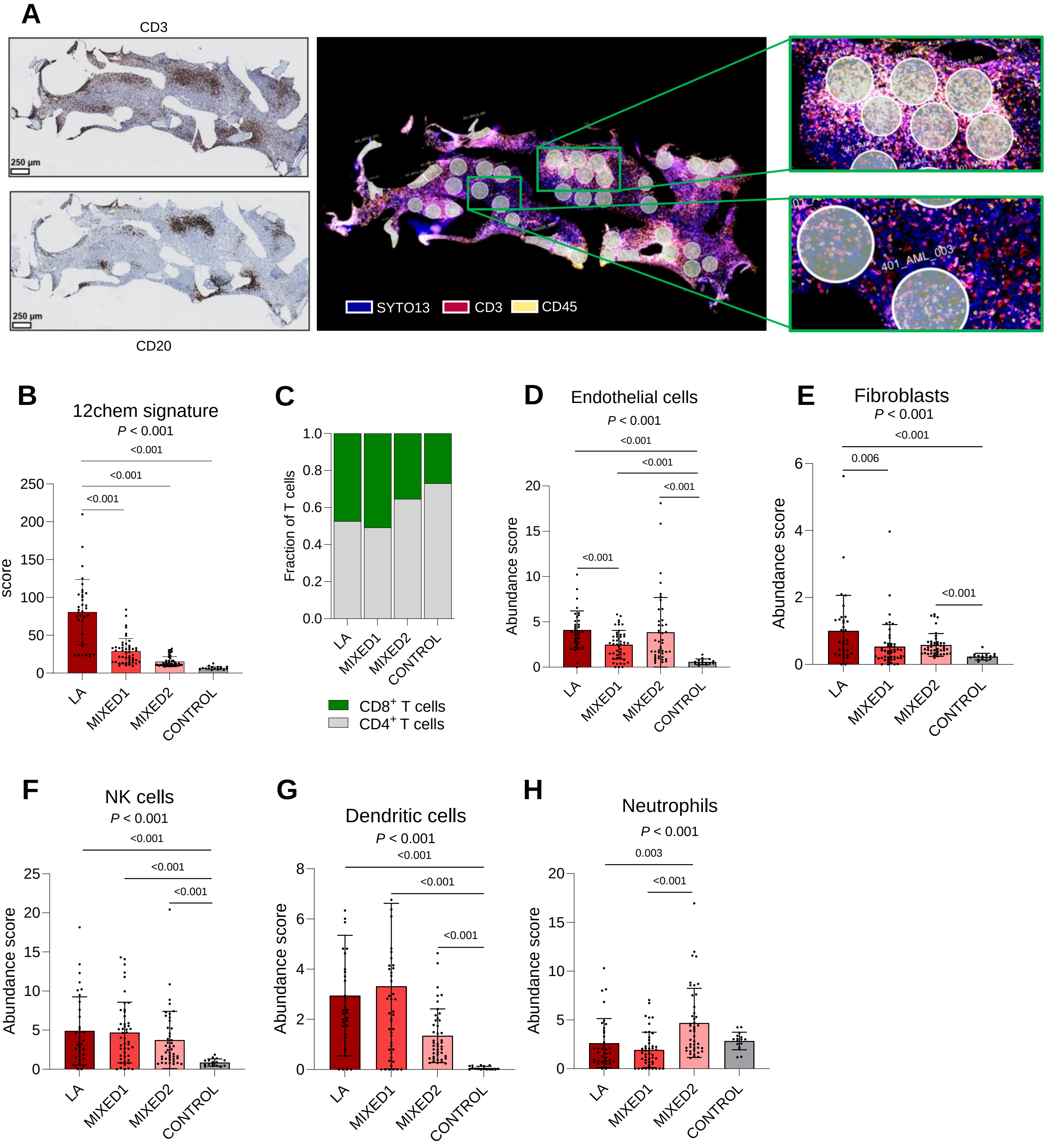

Supplementary Figure 5. Composition of lymphoid aggregates in immune-infiltrated pediatric AML. (A) Representative images of the region of interest (ROI) selection on the GeoMx Digital Spatial Profiling platform. On the left, both the CD3 (T cell) and CD20 (B cell) stains are shown (DAB, both in brown). On the right, magnifications of lymphoid aggregate regions (above) and mixed regions (below) are shown. Selection of ROIs was further aided by overlaying stains of CD34 and CD117. (B) Comparison of the expression of the '12chem' signatures across different region types (Kruskal-Wallis followed by Dunn's multiple comparisons test). (C) Proportions of $CD4^+$ and $CD8^+$ T cells in lymphoid aggregate (LA), mixed, and control regions. (D-H) Deconvoluted absolute abundance of various cell subsets across several region types (Kruskal-Wallis followed by Dunn's multiple comparisons test). In case of multiple p-values, the upper one is associated with the Kruskal-Wallis test, while the lower one(s) reflect(s) the result of Dunn's multiple comparison test.

Supplementary Figure 6

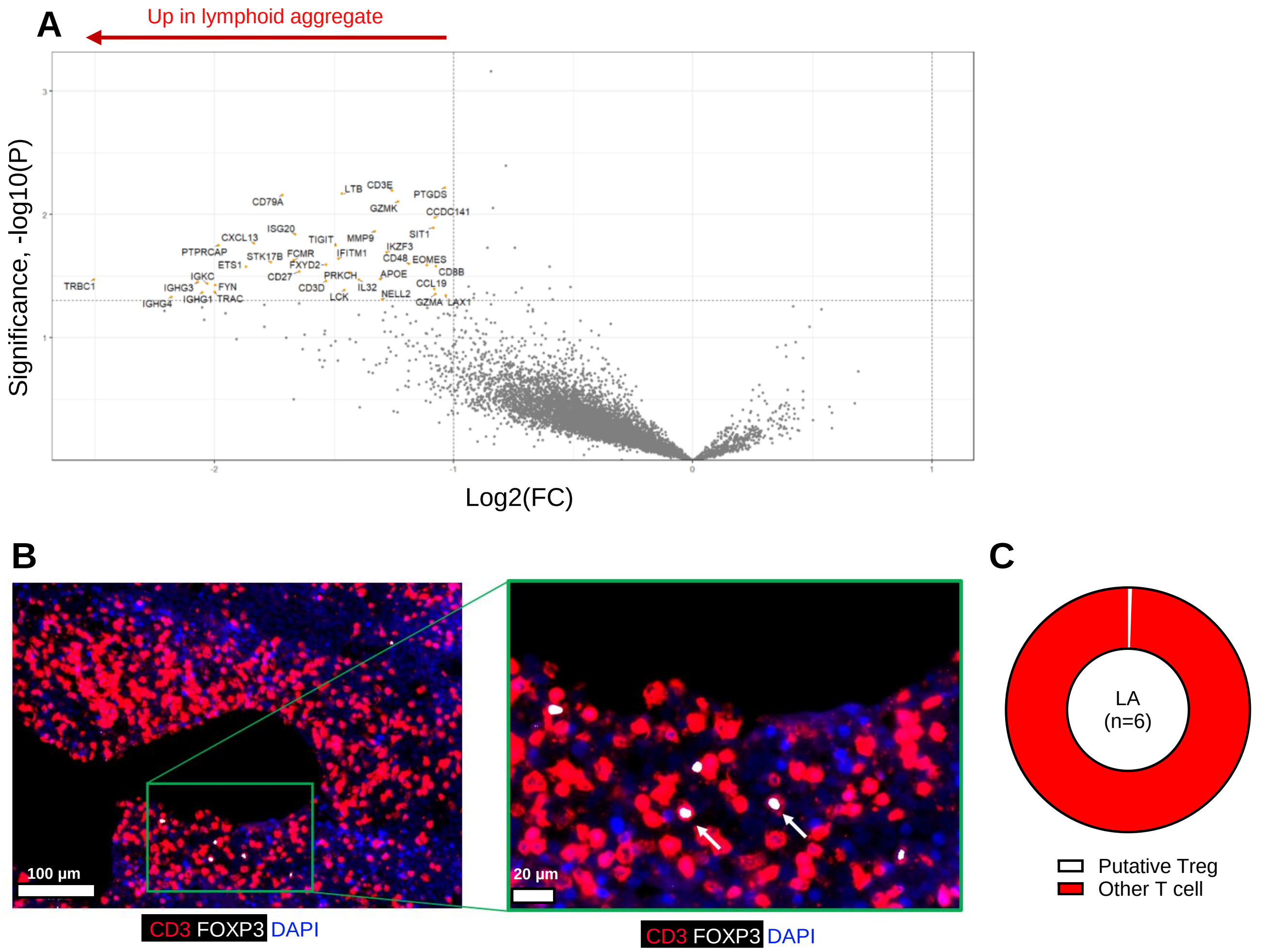

Supplementary Figure 6. Upregulated genes and the presence of regulatory T cells in lymphoid aggregates in immune-infiltrated pediatric AML. (A) Volcano plot of genes differentially expressed in lymphoid aggregates compared to MIXED1 regions, generated using linear mixed-effect modelling. FC: fold change. (B) Representative images of putative Tregs ($CD3^+FOXP3^+$) in lymphoid aggregates ($CD3^+$ T cells). The image on the right reflects a zoom of the region in the green box in the left image. The names below the images reflect the antibodies and the associated colors in the images. (C) Proportion of putative Tregs among all $CD3^+$ T cells in lymphoid aggregates (LA).
